# Assessing social cognition in patients with schizophrenia and healthy controls using the reading the mind in the eyes test (RMET): a systematic review and meta-regression

**DOI:** 10.1017/S0033291723003501

**Published:** 2024-01-04

**Authors:** Fei Deng, Marlys A. Bueber, Yourong Cao, Jeff Tang, Xinyu Bai, Young Cho, Jiwon Lee, Zhuozhi Lin, Qi Yang, Matcheri S. Keshavan, William S. Stone, Min Qian, Lawrence H. Yang, Michael R. Phillips

**Affiliations:** 1 Shanghai Mental Health Center, Shanghai Jiao Tong University School of Medicine, Shanghai, China; 2 University of Nottingham School of Economics (Ningbo China), Zhejiang, China; 3 Guangxi Medical University School of Public Health, Guangxi, China; 4 The Reproductive Hospital of Guangxi Zhuang Autonomous Region, Guangxi, China; 5 New York University, New York, NY, USA; 6 Guangxi Academy of Medical Sciences & The People’s Hospital of Guangxi Zhuang Autonomous Region, Guangxi, China; 7 New York State Psychiatric Institute, New York, NY, USA; 8 Teachers College, Columbia University, New York, NY, USA; 9 Department of Mental Health, Bloomberg School of Public Health, Johns Hopkins University, Baltimore, MD, USA; 10 Ningxia Medical University School of Public Health, Ningxia, China; 11 Harvard Medical School Department of Psychiatry at Beth Israel Deaconess Medical Center, Boston, MA, USA; 12 Department of Biostatistics, Columbia University Mailman School of Public Health, New York, NY, USA; 13 Department of Epidemiology, Columbia University Mailman School of Public Health, New York, NY, USA; 14 Department of Psychiatry, Columbia University, New York, NY, USA

**Keywords:** age, healthy controls, meta-regression, reading the mind in the eyes test, schizophrenia, years of education

## Abstract

The reading the mind in the eyes test (RMET) – which assesses the theory of mind component of social cognition – is often used to compare social cognition between patients with schizophrenia and healthy controls. There is, however, no systematic review integrating the results of these studies. We identified 198 studies published before July 2020 that administered RMET to patients with schizophrenia or healthy controls from three English-language and two Chinese-language databases. These studies included 41 separate samples of patients with schizophrenia (total *n* = 1836) and 197 separate samples of healthy controls (total *n* = 23 675). The pooled RMET score was 19.76 (95% CI 18.91–20.60) in patients and 25.53 (95% CI 25.19–25.87) in controls (*z* = 12.41, *p* < 0.001). After excluding small-sample outlier studies, this difference in RMET performance was greater in studies using non-English *v*. English versions of RMET (Chi [Q] = 8.54, *p* < 0.001). Meta-regression analyses found a negative association of age with RMET score and a positive association of years of schooling with RMET score in both patients and controls. A secondary meta-analysis using a spline construction of 180 healthy control samples identified a non-monotonic relationship between age and RMET score – RMET scores increased with age before 31 and decreased with age after 31. These results indicate that patients with schizophrenia have substantial deficits in theory of mind compared with healthy controls, supporting the construct validity of RMET as a measure of social cognition. The different results for English versus non-English versions of RMET and the non-monotonic relationship between age and RMET score highlight the importance of the language of administration of RMET and the possibility that the relationship of aging with theory of mind is different from the relationship of aging with other types of cognitive functioning.

## Introduction

Individuals with schizophrenia generally exhibit neurocognitive deficits in multiple cognitive domains, including executive function, memory, attention, and problem-solving (Harvey & Rosenthal, 2018; Mesholam-Gately, Giuliano, Goff, Faraone, & Seidman, 2009; Sheffield, Karcher, & Barch, 2018). In addition to neurocognitive impairments, deficits in social cognition – the ability to learn social norms and perceive emotions and other social cues in interpersonal interactions – are commonly seen in individuals with schizophrenia (Green, Horan, & Lee, 2019). The social cognition domain is divided into four sub-domains: emotion processing, social perception, attributional style, and theory of mind (i.e. mentalizing) (Green et al., 2019).

The Measurement and Treatment Research to Improve Cognition in Schizophrenia (MATRICS) Consensus Cognitive Battery (MCCB) (Nuechterlein et al., 2008) is the most widely used battery to comprehensively assess cognition in schizophrenia. However, some authors (Hellemann, Green, Kern, Sitarenios, & Nuechterlein, 2017) have expressed concerns about the cross-cultural validity of the test used to assess social cognition in this battery, the Mayer-Salovery-Caruso Emotional Intelligence Test (MSCEIT) (Mayer, Salovey, & Caruso, 2002). The MSCEIT expects respondents to interpret stories or vignettes about social situations that are unfamiliar to many respondents from non-Western cultures, particularly rural respondents, so it is frequently omitted in studies of cognition in schizophrenia (Deng et al., 2022; Stone et al., 2020).

The reading the mind in the eyes test (RMET) (Baron-Cohen, Wheelwright, Hill, Raste, & Plumb, 2001) is another measure of social cognition used to assess social cognition in schizophrenia. The RMET assesses ‘theory of mind’, a different component of social cognition than the MSCEIT (which assesses the ‘emotion processing’ component of social cognition). It shows respondents the eye region of 36 Caucasian faces and asks them to select one of four accompanying labels that best describes the mental state of the individual pictured. The RMET – which has been translated into more than 20 languages – may be less culture-dependent than MSCEIT; however, there has been no systematic review integrating the results of studies about the use of RMET in schizophrenia, so it is uncertain whether it could be used as an alternative to the MSCEIT in comprehensive measures of cognitive functioning in schizophrenia. Moreover, very few of the available studies that assess the social cognition of individuals with schizophrenia or healthy controls report multivariate analyses that explore the association between RMET results and important covariates, such as age, years of schooling, IQ, race and language of administration – factors that could potentially explain the considerable heterogeneity of RMET performance among participants.

This systematic review identified all studies that use RMET to assess social cognition in separate samples of individuals with schizophrenia or healthy control subjects, not limited to studies that include both these groups. We also conducted a formal assessment of the quality of the reports of these studies. We then compared the RMET results of all identified samples of individuals with schizophrenia with those of all samples of healthy controls and conducted a meta-analysis of data from the subgroup of studies that directly compare RMET results in individuals with schizophrenia and healthy controls. Other study-level meta-regression analyses assessed the relationship of age, level of education, IQ, race, and language of administration (English *v*. non-English) to RMET scores in healthy controls and individuals with schizophrenia.

## Method

### Search

The search algorithm identified some studies that include both patients with schizophrenia and healthy controls, other studies that include patients with schizophrenia with no controls (or with different types of controls), and studies that include healthy controls compared to other types of patients (e.g. patients with autism, bipolar disorder, etc.).

We searched for relevant articles published before 15 July 2020 in three English-language databases (PubMed, Web of Science, and PsycINFO/EBSCO) and two Chinese-language databases (China National Knowledge Infrastructure [CNKI] and Wanfang). The search strategy of the title and abstract of documents included the following terms: (‘RMET’ or ‘Reading the Mind in the Eyes’ or ‘Reading the Mind in the Eye’) OR (‘schizophrenia’ AND ‘eye test’). The detailed search strategy for each database is shown in the online Supplementary materials. Reference lists of the papers meeting eligibility criteria were individually searched to identify additional studies.

### Eligibility criteria

Original research studies using the 36-item version of RMET that report the crude RMET score (i.e. the number of correctly classified pictures) of patients with schizophrenia or healthy controls were included. Studies were excluded if the individuals with schizophrenia or healthy controls were under 18 or had a history of mental retardation, autism spectrum disorder, epilepsy, brain injury, brain disease, substance use disorder, or other mental disorders. To reduce the heterogeneity between the samples of individuals with schizophrenia included in the analysis, studies with samples that combined different psychotic disorders (for example, schizophrenia and schizoaffective disorder, delusional disorder or affective disorders with psychotic symptoms) were only included if they provided separate results for the subsample of individuals with schizophrenia (results for non-schizophrenia subsamples in these studies were not included in this review).

### Selection of studies

Several reviewers (MAB, YRC, JT, XB, YC, JL, ZL, and QY) screened the titles and abstracts of studies identified in the electronic searches of the databases to decide whether they potentially met the eligibility criteria. Two independent reviewers had to agree on the classification of each article; disagreement was resolved by the senior author (FD). Full-text versions of the potentially eligible articles were then retrieved and independently reassessed by two reviewers (MAB, YRC, JT, XB, YC, JL, and ZL) to ensure that they met the inclusion criteria; disagreements about the final selection were resolved through discussion with the senior author (FD).

### Data extraction

The following information about each selected article was entered in a pre-designed table:

- study characteristics (first author, title, journal, year of publication, and language of publication);
- type of study population(s) (patients with schizophrenia only, healthy subjects only, both patients with schizophrenia and healthy controls, or healthy controls compared to patients with other diagnoses);
- characteristics of the study population (country of test administration, source of participants, sampling method, inclusion or exclusion criteria of the study, diagnostic criteria employed to screen subjects, sample size);
- characteristics of included participants (gender, age, years of schooling, urban or rural residence, ethnicity, treatment status [of individuals with schizophrenia]);
- language of RMET test;
- method of administering RMET (interviewer-completed, paper and pencil self-completion, computer-based self-completion, or online self-completion);
- RMET test results (mean and s.d. of RMET scores and results of multivariate analyses if available) and
- (only from papers that include patients with schizophrenia and healthy controls) crude and adjusted results of comparing RMET scores between patients with schizophrenia and healthy controls.

Two independent reviewers (MAB, YRC, JT, XB, YC, and QY) extracted data for each included study; the senior author (FD) made a final determination in cases where the two reviewers disagreed.

### Quality assessment

The quality assessment scale developed for this study included the 11 items listed in Table 1. The list combined adapted versions of items used in the STROBE (Strengthening the Reporting of Observational Studies in Epidemiology) statement (von Elm, Altman, Egger, Pocock, & Gøtzsche, 2008) with items based on the authors’ experience administrating the RMET test. Each item was coded as ‘1’ or ‘0’ based on whether the paper fulfilled the criteria specified in the item. Thus, the theoretical range of the total quality score was 0 to 11. We categorized the overall quality score based on these scores: 0–4=‘poor’,5–7=‘fair’, 8–11 = ‘good’. Two reviewers independently assessed the quality of each paper (MAB, YRC, JT, XB, YC, JL, and ZL); disagreements in any of the 11 item scores for each paper were resolved by the senior author (FD).

### Analysis

The *T* test was used to compare the study quality score between study samples of patients with schizophrenia and healthy controls and between samples using different language versions (English *v*. non-English). The mean RMET score(s), the mean of the number of correctly classified pictures in each group of respondents, was used as the outcome variable for each study. Both regular random-effect models and DerSimonian-Laird random-effect models were used to estimate the pooled score of RMET separately in patients with schizophrenia and healthy controls. The DerSimonian-Laird random-effect model is particularly useful when pooling samples that have heterogeneous results (DerSimonian & Laird, 1986). The *Z* test was used to compare pooled estimates of RMET scores in patient samples and healthy control samples.

A random-effects model was used to compare the standard mean difference of RMET scores between individuals with schizophrenia and healthy controls in the studies that included both types of respondents because the effect size estimates were heterogeneous. In this analysis, effect sizes for each group were weighted using the inverse variance method. Q statistics, which follow a chi-square distribution, were used to assess standardized within-study differences. The heterogeneity of estimates across studies was assessed using *I*^2^, which represents the proportion of the variance in the estimates due to heterogeneity (Higgins, Thompson, Deeks, & Altman, 2003). A funnel plot was used to evaluate potential publication bias, and Egger’s test assessed the small-size effect (Egger, Davey, Schneider, & Minder, 1997). We also used other methods to determine publication bias recommended by Carter, Schonbrodt, Gervais, and Hilgard (2019): trim-and-fill imputation, precision-effect test (PET), and precision-effect estimate with standard error (PEESE). Subgroup analysis evaluated the possible influence of the language of the administered RMET on the outcome.

Both univariate and multivariate meta-regression assessed the association of age, years of schooling, IQ, race, and language of administration with the RMET score in individuals with schizophrenia and healthy controls. The meta-regression equations were estimated using two different methods: restricted maximum likelihood (Viechtbauer, 2005) and bootstrap (Davison & Hinkley, 1997).

The mean age in the 180 samples of healthy controls that provided age data covered a wide range (from 18.7 to 71.7 years old), making it feasible to conduct a meta-regression with spline construction of age to identify a potential none-monotonic relationship between age and the RMET score in both univariate and multivariate analyses. All ages from 25 to 45 were fitted as the knot value, and the model with the lowest AIC was considered the best-fitted model.

Data were analyzed using the STATA 17.0 version.

### Registration

The protocol of this systematic review was registered on PROSPERO on 30 November 2020 before starting the title and abstract screening of the electronically identified studies (registration ID: CRD 42020216401).

## Result

### Selection of studies

**Figure 1.**
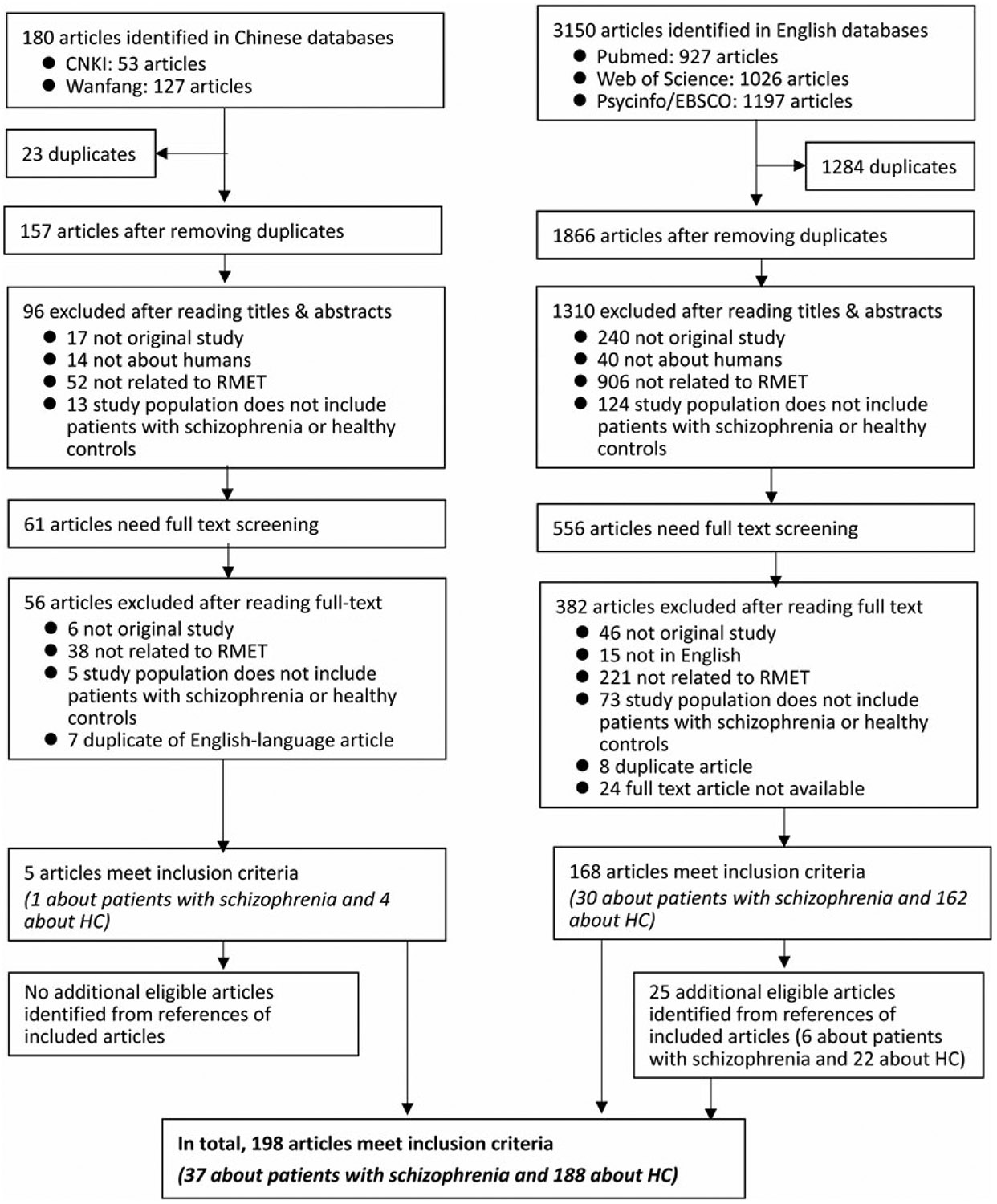
Flowchart of the identification of articles.

As shown in Fig. 1, the titles and abstracts of 1886 articles identified in English-language databases and 157 articles identified in Chinese-language bases were screened to identify potentially eligible papers. Based on this preliminary screening by two independent reviewers, the kappa values for potential inclusion were 0.72 for English articles and 0.77 for Chinese articles. The full text of potentially eligible articles (556 in English and 61 in Chinese) was then reviewed by two independent reviewers; the kappa value for inclusion based on this final screening was 0.61 for English articles and 0.52 for Chinese articles. After screening the electronically identified articles and identifying additional articles from the reference lists of selected articles, 198 studies were included in the analysis, 5 in Chinese and 193 in English. These 198 studies included 41 separate samples of patients with schizophrenia (with a total of 1836 patients) and 197 separate samples of healthy controls (with a total of 23 976 individuals). Only 26 (13.1%) of the studies (with 1455 patients with schizophrenia and 1087 healthy controls) directly compared RMET results in individuals with schizophrenia and healthy controls. Among the 41 samples of patients with schizophrenia, 8 (19.5%) used the English-language version of RMET, 3 (7.3%) used the Chinese-language version, 29 (70.7%) used other language versions, and 1 (2.4%) used two language versions (English and Korean). Among the 197 samples of healthy controls, 75 (38.1%) used the English-language version of RMET, 7 (4.1%) used the Chinese-language version, 110 (55.8%) used other language versions of RMET, 1 used two language versions (English and Korean), and the language version used in 4 (2.0%) study samples was unknown. The detailed characteristics of these studies are shown in Table 2.

### Quality evaluation

Among the 41 samples of patients with schizophrenia included in the 198 papers, one reported a mean RMET score without an accompanying standard deviation (or standard error), and five did not include data on the mean educational level of participants. Among the 197 samples of healthy controls included in the 198 papers, four reported mean RMET scores without an accompanying standard deviation, 17 did not include data on the mean age of participants, and 99 did not include data on the mean educational level of participants.

The items used to assess study quality are shown in Table 1, and the results of the quality assessment of the 198 included studies are shown in the last column of Table 2. The total quality score (theoretical range 0–11) varied from 2 to 10. The mean (s.d.) quality score of all papers was 5.9 (1.4); 28 (14.1%) papers were classified as ‘poor quality’ (score 0–4), 148 (74.7%) as ‘fair quality’ (score = 5–7), and 19 (9.6%) as ‘good quality’ (score = 8–11). Among the 11 separate items, only five items were present in more than 75% of studies (items 1, 2, 6, 9, and 11 shown in Table 1). Four items were *absent* in more than 75% of the studies: description of study setting (item 3), rationale for sample size (item 5), number of study drop-outs (item 8), and adjustment of RMET results (item 10).

When assigning the quality assessed for the paper as a whole to each of the included samples in each paper, the overall mean quality score for the 238 samples was 6.0 (1.5); 22 (13.5%) poor quality, 173 (72.7%) fair quality, and 33 (13.9%) good quality. The mean quality score of the 41 samples of patients with schizophrenia was significantly higher than that of the 197 samples of healthy controls [6.7 (1.8) *v*. 5.9 (1.4); *t* = 3.41, *p* < 0.001]. The mean quality score in the 149 samples administered non-English versions of RMET was significantly higher than that of the 83 samples administered the English version of RMET [6.3 (1.4) *v*. 5.6 (1.5); *t* = 3.13, *p* = 0.002].

### Pooled RMET scores of patients with schizophrenia and healthy controls

**Figure 2.**
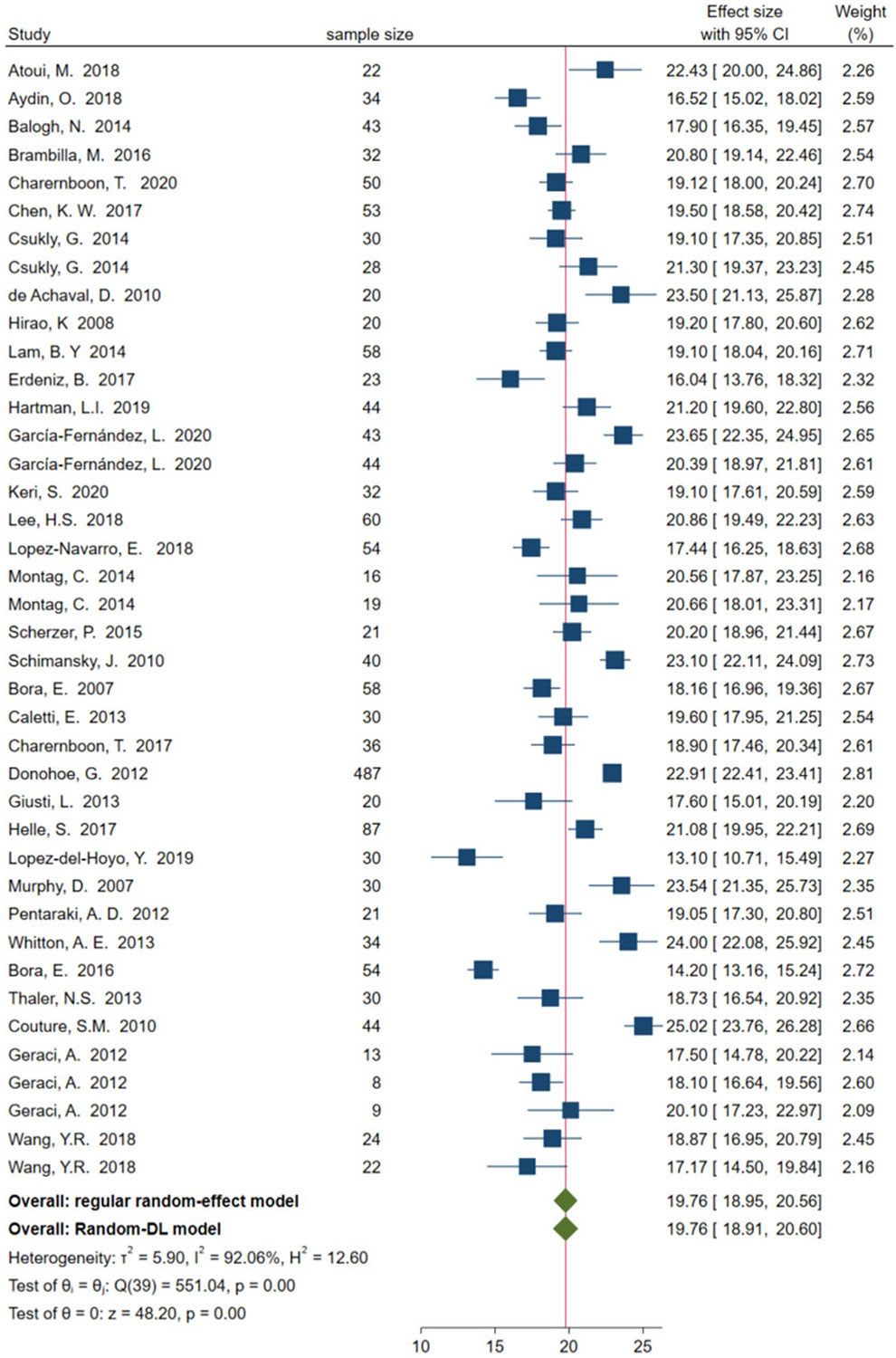
Pooled estimates of mean RMET scores in samples of patients with schizophrenia (including 40 separate samples reported in 36 different papers with a total sample size of 1823 individuals with schizophrenia).

**Figure 3.**
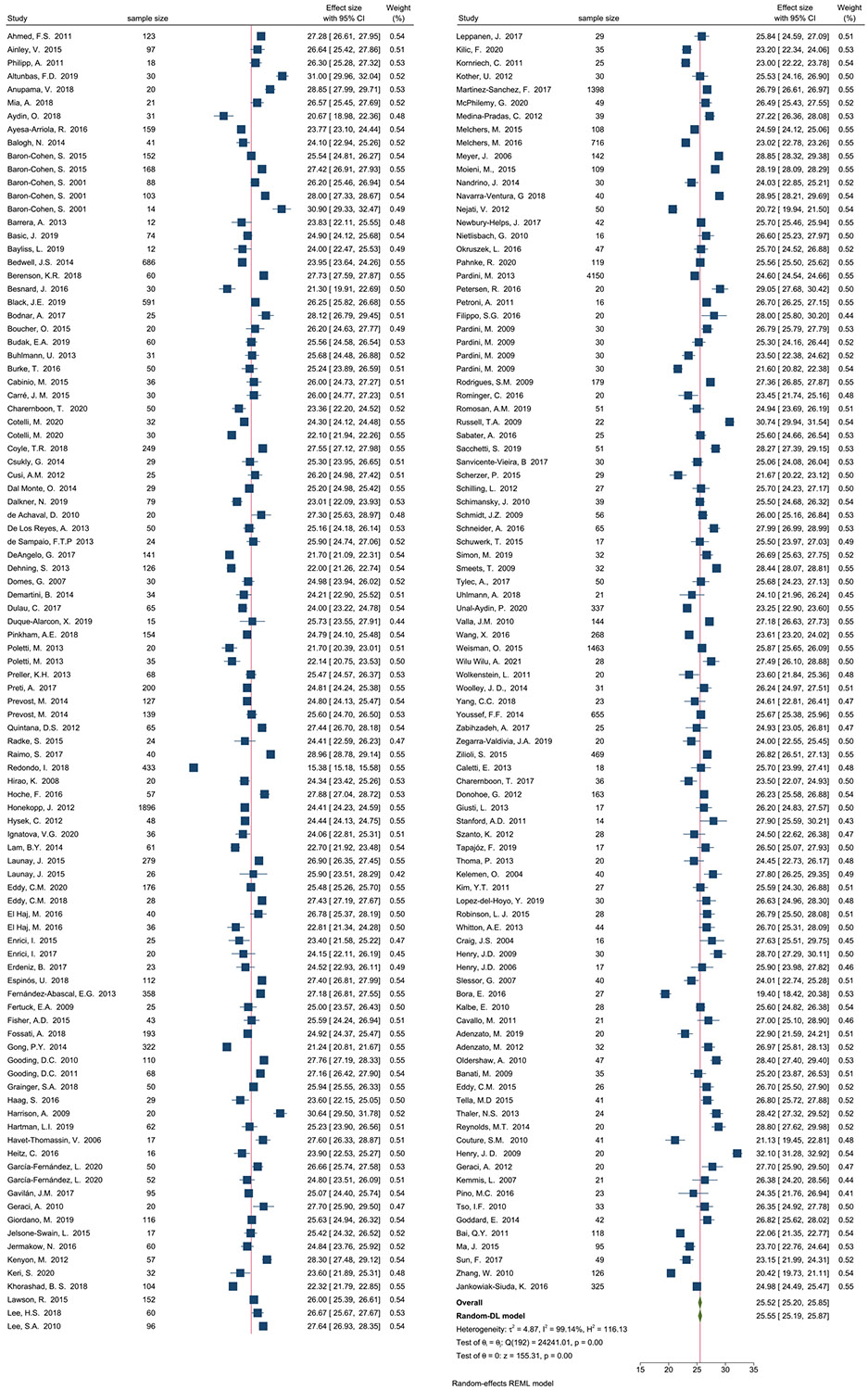
Pooled estimates of mean RMET scores in samples of healthy controls (including 193 separate samples reported in 185 different papers with a combined sample size of 23 619 individuals).

The pooled RMET scores in patients with schizophrenia and healthy controls are shown in Figs 2 and 3. Based on the results of 1823 patients reported in 40 separate study samples that provided both the mean and standard deviation of RMET scores, the pooled estimate for the RMET score in patients was 19.76 (95% CI 18.91–20.60). Based on the results of 23 619 healthy controls reported in 193 separate study samples that provided both the mean and standard deviation of RMET scores, the pooled RMET score in healthy controls was 25.53 (95% CI 25.19–25.86) – significantly higher than that in the patient samples (*z* = 12.41, *p* < 0.001).

### Direct comparison of RMET results between patients with schizophrenia and healthy controls

**Figure 4.**
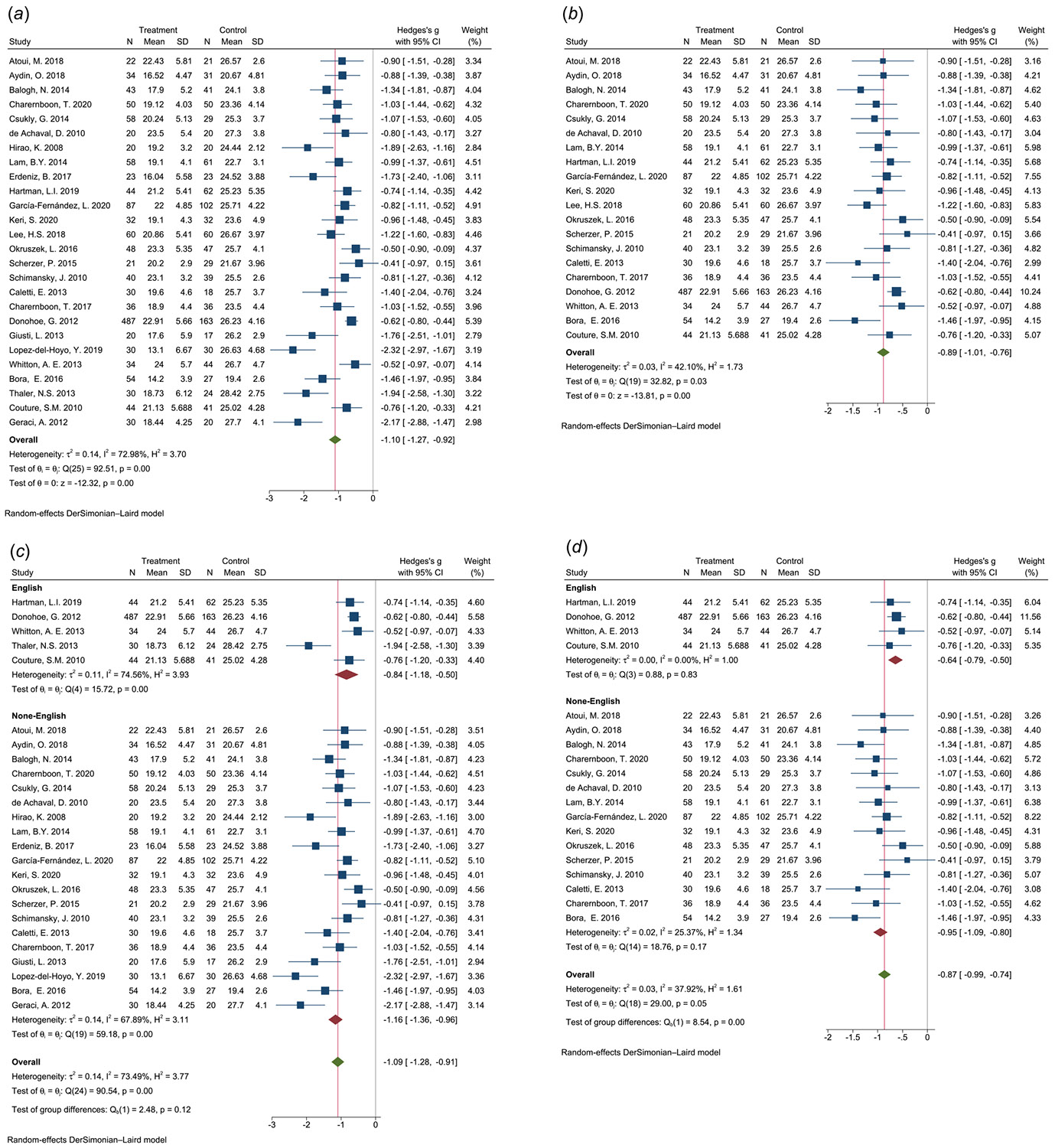
Forest plot of the standardized mean difference of RMET score between different types of respondents. **Panel A:** Comparison of individuals with schizophrenia and healthy controls (26 studies). **Panel B:** Comparison of individuals with schizophrenia and healthy controls after removing the outliners (20 studies). **Panel C:** Comparison of individuals with schizophrenia and healthy controls stratified by the version of RMET (English v. non-English) (26 studies). **Panel D:** Comparison of individuals with schizophrenia and healthy controls stratified by the version of RMET (English v. non-English) after removing the outliners (20 studies).

Among the 26 studies that directly compared mean RMET scores of patients with schizophrenia and healthy controls, only one study (Scherzer, Achim, Leveille, Boisseau, & Stip, 2015) did not find a statistically significant difference between the two groups; all other studies reported significantly lower mean RMET scores in the patient group. As shown in Fig. 4A, the pooled standard mean difference for the 26 studies estimated by a random-effect meta-analysis model indicated that the RMET scores in patients with schizophrenia were 1.10 standard deviations lower than the RMET scores in healthy controls (*z* = −12.32, *p* < 0.001).

**Figure 5.**
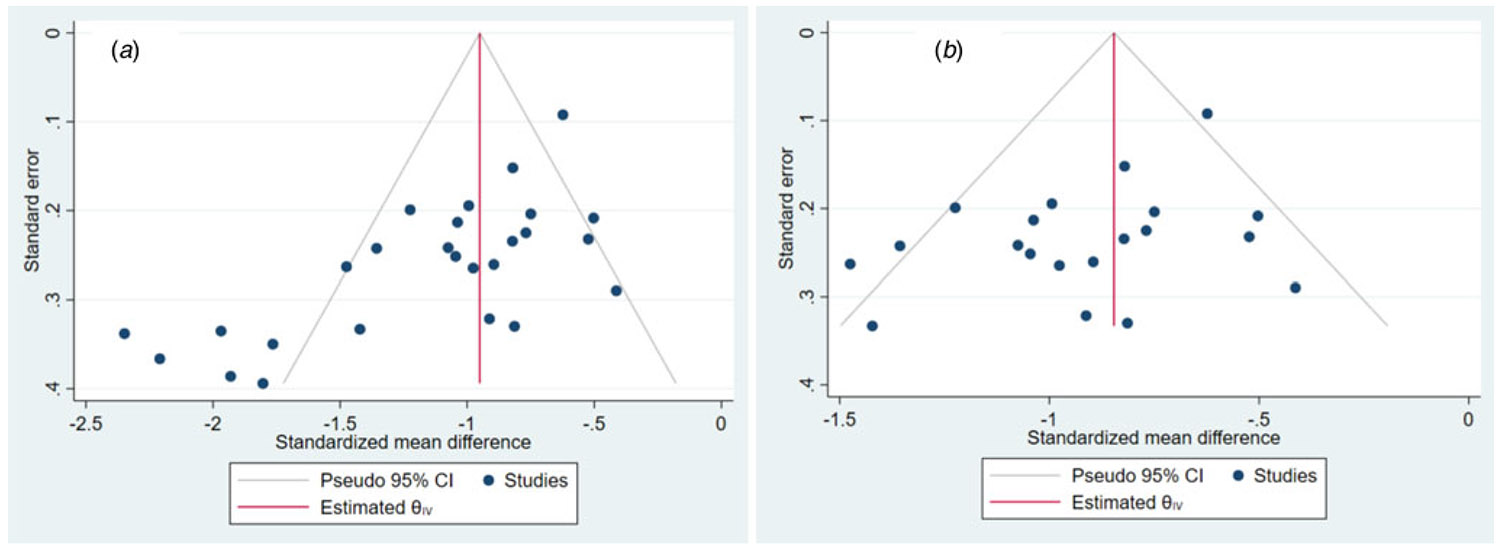
Funnel plots of results of meta-analyses. **Panel A:** Results of all 26 studies comparing individuals with schizophrenia and healthy controls **Panel B:** Results of 20 studies that remain after removing studies with outlier results.

There was substantial heterogeneity in the estimated effect sizes of the 26 studies: the *I*^2^ value was 73.0%, and the corresponding *Q* statistic value was 92.5 (*p* < 0.001). The funnel plot for the 26 studies (Fig. 5A) identifies the main reason for this heterogeneity; the plot is imbalanced because the six smallest studies (total sample sizes ranging from 37 to 60) have the six largest effect sizes. Thus, the potential for publication bias is high, a finding supported by the results of Egger’s test (*z* = −4.53, *p* < 0.001). None of the statistical methods recommended to reduce the effect of publication bias due to the six outlier studies (trim-and-fill imputation, PET, and PEESE) effectively reduced the bias, so we conducted a sensitivity analysis by re-assessing the results after removing the data from the six studies. After removing these six outliners, the funnel plot for the remaining 20 studies is balanced (Fig. 5B); the pooled standardized mean difference is reduced but still statistically significant (SMD = 0.89; *z* = −13.81, *p* < 0.001); and the *I*^2^ value is reduced to 42.1% and the corresponding Q-test value was 32.8 (*p* = 0.03) (Fig. 4B).

Among the 26 studies that directly compared patients with schizophrenia to healthy controls, five studies used the original English version of RMET (Baron-Cohen et al., 2001), one study used the English version in half of the participants and a Korean version in the other half, and 20 studies used translated versions of RMET (Turkish, Hungarian, Italian, and Spanish were each used in three papers; Thai was used in two papers; and Chinese, French, German, Japanese, Lebanese, and Polish were each used in a single paper). Based on the stratified analyses (Fig. 4C), the pooled SMD was greater in the 20 studies using non-English versions (SMD = −1.16, z = 11.22, *p* < 0.001) than in the five studies using the English version (SMD = −0.84, *z* = 3.28, *p* = 0.001) and heterogeneity was greater in studies using the English version (*I*^2^ = 74.6%, *p* < 0.001) than in studies using non-English versions (*I*^2^ = 67.9%, *p* < 0.001). The SMD was not significantly different between these language-based subgroups when all 25 study samples were included in the analysis (Chi [Q] = 2.48, *p* = 0.12). However, after excluding the six smallsample outlier studies (Fig. 4D), the SMD in the remaining 15 non-English RMET studies was significantly greater than the SMD in the remaining four English RMET studies (−0.95 *v*. −0.64, Chi[Q] = 8.54, *p* < 0.001), but the four remaining studies using the English version were less heterogeneous than the 15 remaining studies that used non-English versions (*I*^2^ = 0.0% in the four English RMET studies, and *I*^2^ = 37.9% in the 15 non-English RMET studies).

### Meta-regression on the covariates

**Figure 6.**
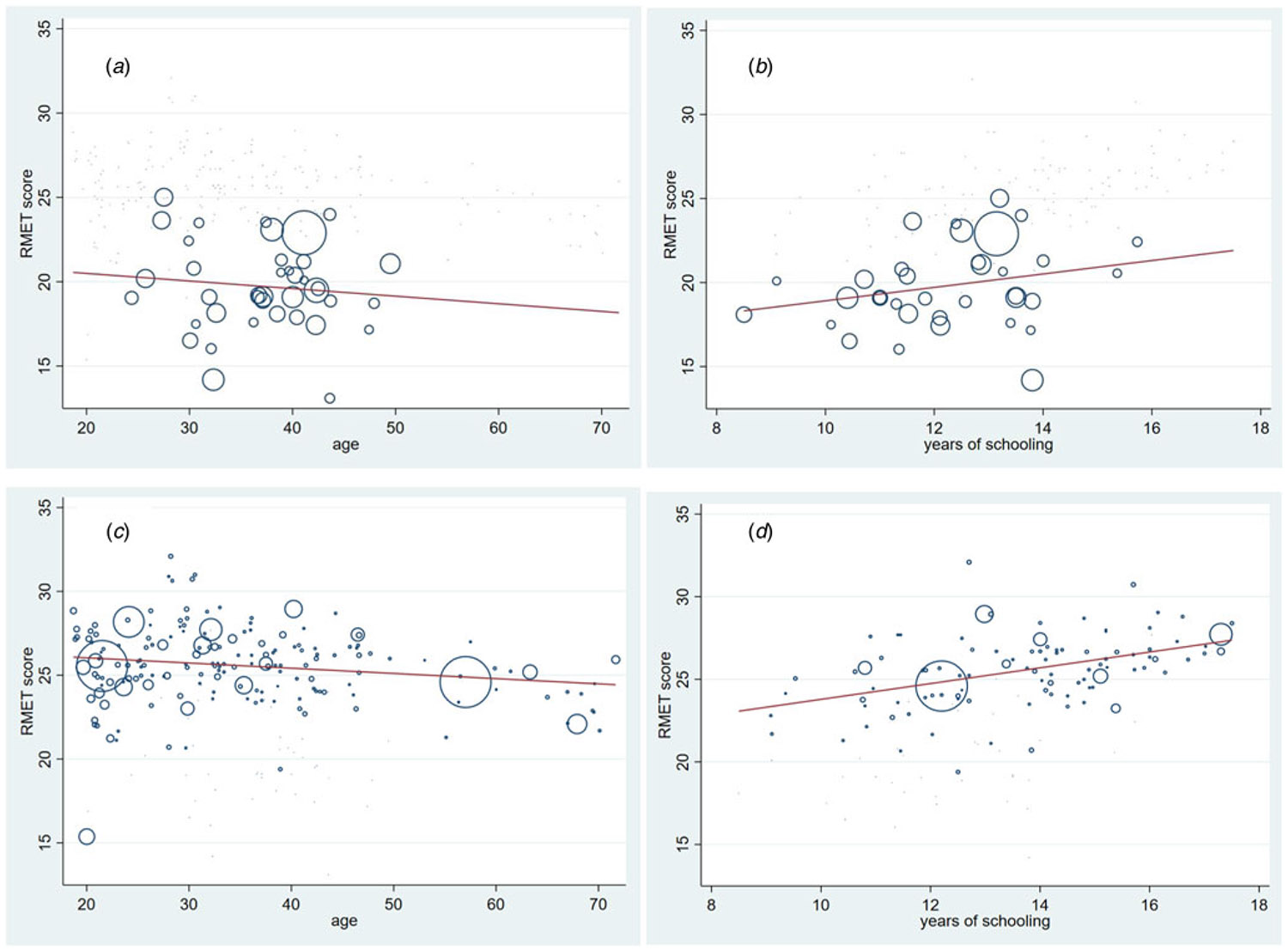
Association of age and years of schooling with RMET score in different respondents based on univariate meta-regression. **Panel A:** Association of age and RMET score in individuals with schizophrenia in 40 study samples. **Panel B:** Association of years of schooling and RMET score in individuals with schizophrenia in 35 study samples. **Panel C:** Association of age and RMET score in healthy controls in 180 study samples. **Panel D:** Association of years of schooling and RMET score in healthy controls in 99 study samples.

There were 36 studies with 40 distinct samples of individuals with schizophrenia (combined sample size = 1823) that provided both the mean age of the sample and the mean and standard deviation of the RMET scores; 29 of these studies included 35 distinct samples with schizophrenia (combined sample size = 1620) that also provided the mean years of schooling of the sample. These data made it possible to conduct three separate regression analyses that included age, schooling, and both age and schooling as independent variables. Each regression equation was estimated using two methods: restricted maximum likelihood and the bootstrap method. As shown in Table 3, when the regression only had age as an independent variable (Model 1, Fig. 6A), the RMET score decreased with increasing age, but this decreasing trend was not statistically significant (*β*; = −0.045, *p* = 0.516). When the regression only included years of schooling as an independent variable (Model 2, Fig. 6B), the RMET score increased with increasing years of schooling, but this increasing trend was not statistically significant (*β* = 0.399, *p* = 0.149). Multivariate meta-regression using both mean age and mean years of schooling as independent variables (Model 3) also showed the negative relationship between RMET score and age (*β* = −0.032, *p* = 0.635) and the positive relationship between RMET score and years of schooling (*β* = 0.418, *p* = 0.140) in patients with schizophrenia, but neither of these associations was statistically significant. The results using the two estimation methods were quite similar, but the *p* values for the coefficients related to years of schooling are substantially smaller when using the bootstrap method.

A parallel meta-regression analysis of healthy control subjects used the results from 180 distinct samples (combined sample size = 21 494) that included data on the mean age of respondents; 98 of these samples (combined sample size = 7946) also included data on the mean years of schooling of respondents. In these analyses, the regression that only included age as an independent variable (Model 1, Fig. 6C) identified a statistically significant decrease in RMET scores with increasing age (*β* = −0.031, *p* = 0.020); the regression that only included years of schooling as an independent variable (Model 2, Fig. 6D) found a statistically significant increase in RMET scores with increasing years of schooling (*β* = 0.477, *p* < 0.001); and the multivariate meta-regression that included both age and years of schooling as independent variables (Model 3) found that increasing years of schooling remained significantly associated with increasing RMET scores (*β* = 0.423, *p* < 0.001), but the relationship of increasing age with decreasing RMET scores was no longer statistically significant (*β* = −0.026, *p* = 0.126). In this case, the only difference in the two estimation methods was a smaller *p* value for age in Model 3.

The differences in the association of age and education with RMET scores between the patient samples and healthy control samples may be related to the number of distinct samples available for the different analyses. For example, in the regressions using age as an independent variable, the coefficient for the 40 patient samples was substantially greater than that for the 180 healthy control samples (*β* = −0.045 *v*. *β* = −0.031), but the relationship of decreasing RMET scores with increasing age in the healthy control samples was statistically significant, whereas that in the patient samples was not. Similarly, in the multivariate meta-regression analysis, the coefficient for the adjusted relationship of years of schooling in the 35 patient samples (*β* = 0.418) is essentially identical to that for the 99 healthy control samples (*β* = 0.423). However, the relationship of increasing RMET scores with increasing years of schooling is not statistically significant for the patient groups (*p* = 0.140), while it is statistically significant for the healthy control groups (*p* < 0.001).

In the multivariate meta-regression, the larger negative coefficient for age in the patient samples compared to that in the healthy control samples (*β* = −0.032 *v*. *β* = −0.026) suggests that after adjusting for years of schooling, the annual rate of decline in social cognitive functioning (as assessed by RMET) in patients with schizophrenia is 23% ([0.032–0.026]/0.026) faster than that in healthy controls.

We also considered IQ and race (Caucasian *v*. other) potential covariates. However, only 6 of the 41 studies with patient samples provided IQ, and only 7 of the studies provided data on race, so it was not feasible to conduct a meta-regression in the patient samples. There were, however, 26 studies with samples of healthy controls that provided IQ (19 of which also provided data on years of schooling) and 21 studies with samples of healthy controls that provided data on race (9 of which also provided data on years of schooling). In the univariate meta-regression of the RMET score and IQ, IQ had a non-significant positive association with the RMET score (*β* = 0.046, *p* = 0.413); in the multivariate meta-regression (RMET scores *v*. IQ and years of schooling), the positive association of RMET with years of schooling was statistically significant (*β* = 0.492, *p* = 0.045) while that with IQ remained non-significant (*β* = −0.066, *p* = 0.319). In the univariate meta-regression of RMET score and race, the proportion of Caucasians in the sample had a non-significant negative association with the RMET score (*β* = −0.152, *p* = 0.907) while in the multivariate meta-regression (RMET scores *v*. race and years of schooling) the positive association of RMET scores with years of schooling was no longer statistically significant (*β* = 1.199, *p* = 0.064) and the proportion of Caucasian subjects in the sample had a non-significant positive association with RMET scores (*β* = 3.296, *p* = 0.151).

### Assessment of non-monotonic relationship between age and RMET score in healthy controls

**Figure 7.**
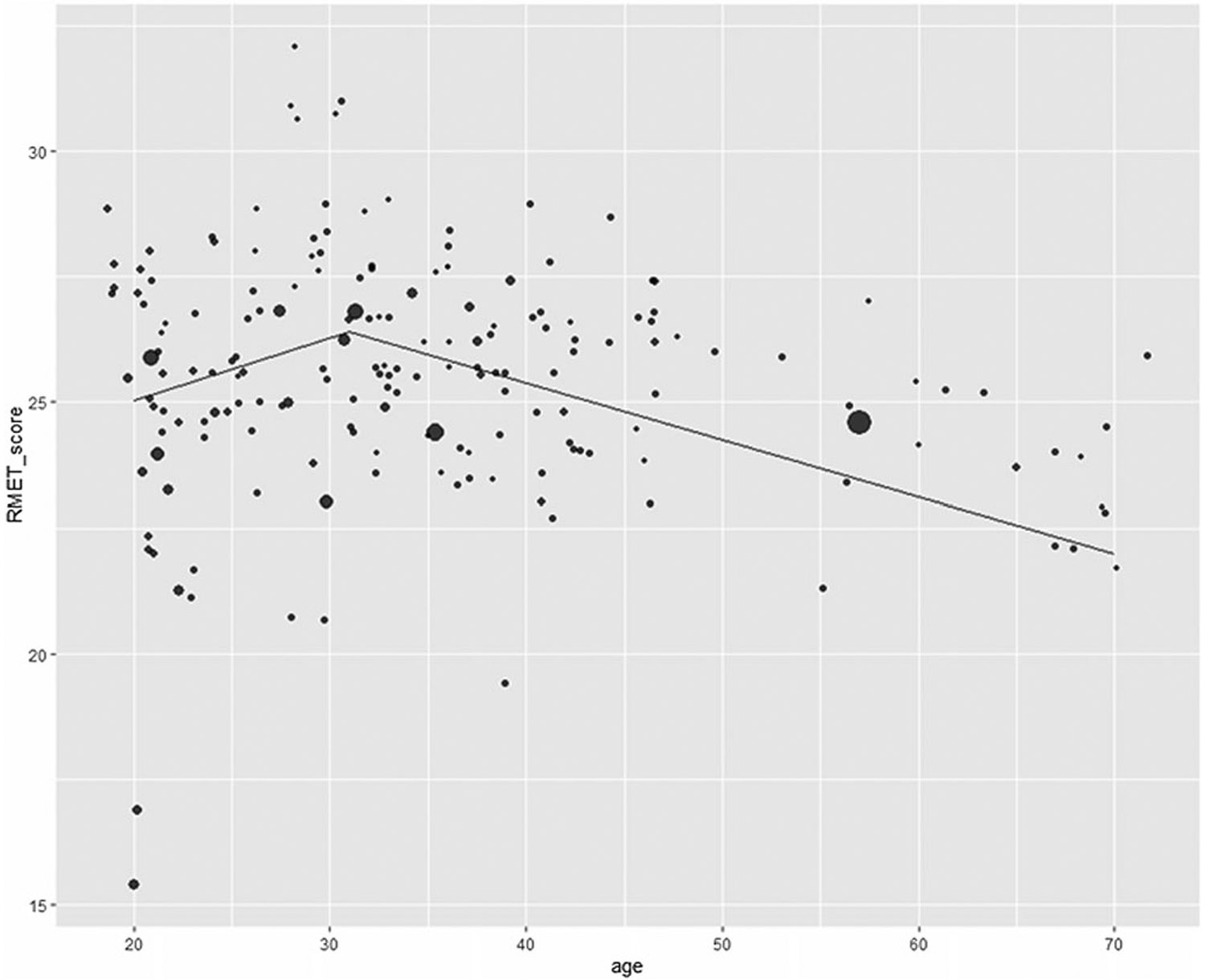
Relationship between age and RMET score in healthy controls using univariate linear regression with spline construction in 180 separate study samples.

The mean age of individuals in the 180 samples of healthy controls that included data on age ranged from 18.7 to 71.7, making it possible to assess a potential non-linear relationship of age with RMET scores using linear regression with spline construction. Assessing potential knots from 25 to 45 years of age, we identified 31 years of age as the point of inflection (i.e. the knot with the lowest AIC) for both univariate regression (only including age, AIC = 792.2) and multivariate analysis (including age and years of schooling, AIC = 422.7). As shown in Table 4 and Fig. 7, in the univariate analysis, the RMET score increased with age before age 31 (*β* = 0.123, *p* = 0.008) and declined with age after age 31 (*β* = −0.074, *p* < 0.001). In the multivariate model (Table 4), after adjusting for years of schooling (which was significantly associated with RMET score), the RMET showed a significant increase with age before age 31 (*β* = 0.179, *p* = 0.048) and a statistically significant decline with age after age 31 (*β* = −0.048, *p* = 0.011).

## Discussion

This review identified 198 studies that used RMET to assess social cognition in 41 separate samples of patients with schizophrenia and 197 separate samples of healthy controls. The pooled mean RMET score of the 1823 patients and 23 619 healthy controls included in these studies was much lower in patients than in healthy controls (19.8 [18.9–20.6] *v*. 25.5 [25.2–25.9], *z*= 12.41, *p* < 0.001). Meta-analysis of the results of 26 studies that directly compared RMET scores in patients with schizophrenia and healthy controls found that the pooled mean of patients’ scores was more than one SMD lower than the pooled mean score of healthy controls. Significant publication bias was identified among these studies (studies with smaller sample sizes were more likely to report larger SMD between the two groups), but the differences between groups remained significant after removing the six outlier studies with potential publication bias. These results confirm previous findings that patients with schizophrenia are suffering from substantial deficits in theory of mind.

Subgroup analyses indicated that after excluding the outlier studies the difference in RMET performance between patients with schizophrenia and healthy controls was greater in studies using non-English versions of RMET than in those using the original English version (Chi [Q] = 8.54, *p* < 0.001). The reasons for this difference are unclear. All of the studies used the same sets of pictures (with Caucasian subjects), so it is likely (though not certain) that some of the respondents administered non-English versions of RMET were less racially and ethnically similar to the individuals in the stimulus pictures than respondents administered the English version of RMET. The difficulty patients have in identifying emotions in the RMET may be magnified when presented with pictures of persons with an ethnicity different from their own, resulting in a greater assessed deficit compared to healthy controls in studies that use non-English versions of RMET. One previous study reporting that children perform better when recognizing the emotions of their own-race faces than other-race faces (Segal, Reyes, Moulson, & Gobin, 2019) supports this hypothesis. Further research with RMET using non-Caucasian pictures is needed to clarify this issue.

The results for patients and healthy controls were quite heterogeneous, so we used meta-regression methods to explore the relationship between mean RMET performance, mean age, and mean level of education in patient samples and, separately, in healthy control samples. In the univariate analyses, age was negatively related to the RMET score and educational level was positively related to the RMET score in both the patient samples and the healthy control samples, but the results were only statistically significant for the healthy control samples, possibly because of the much smaller number of patient samples available for analysis. A separate meta-regression with spline construction in the healthy control samples found that RMET scores increased with age before age 31 and decreased with age after age 31. (The much smaller number of samples of patients with schizophrenia and the smaller range in the mean age of these samples made it infeasible to conduct a spline construction meta-regression using the patient samples.) These relationships persisted in the multivariate analysis (including age and years of schooling as covariates), though the effect of age was attenuated after adjustment for years of schooling.

Previous findings about the relationship between age and RMET scores have been inconsistent. Dodell-Feder, Ressler, and Germine (2020) used online interviews to assess RMET in 40 248 participants 10–70 years of age and found that RMET scores increased with age up until 65. Cabinio et al. (2015) reported unchanging RMET scores in healthy respondents 20–70. Two cross-sectional studies (Javkowiak-Siuda et al., 2016; Slessor, Phillips, & Bull, 2007) comparing RMET performance in persons over 65 to that of persons under 35 found that the older participants had significantly lower RMET scores. Finally, Pardini and Nichelli (2009), Deng et al. (2022), and Lee, Nam, and Hur (2020) reported that RMET performance started to decline in the fifth decade of life, at age 60 and age 66, respectively. Several hypotheses have been proposed to explain increasing deficits in theory of mind with aging. Slessor et al. (2007) suggested that deficits in theory of mind are manifestations of general impairment in the ability to decode cues. Some researchers suggest that the decline of theory of mind is mediated by impairment in other cognitive domains, such as executive function, information processing speed (Charlton, Barrick, Markus, & Morris, 2009), destination memory (El Haj, Raffard, & Gély-Nargeot, 2016), and verbal intelligence (Slessor et al., 2007). Furthermore, neuroimaging studies report that declines in RMET score with aging are correlated with decreasing volume in the bilateral precentral gyrus, bilateral posterior insula, left superior temporal gyrus, and left inferior frontal gyrus (Cabinio et al., 2015). Our systematic review of 198 studies that administered RMET to 180 separate samples of healthy subjects is the first study to identify a non-monotonic relationship between RMET score and age, suggesting that individuals accumulate knowledge and skills of theory of mind until they reach early middle age (32 years of age), and then their theory of mind performance gradually declines with normal aging. This raises the possibility that the neurodevelopmental trajectory of social cognition is more prolonged than that of other types of cognition (i.e. continuing to develop as the individual’s social world expands during adolescence and young adulthood) and, thus, can be disrupted at later ages by serious mental illnesses like schizophrenia.

In this review we found that the association of years of schooling with RMET scores was more robust than the association of age with RMET scores, but there has been much less research about the role of education in the development of theory of mind. Khorashad et al. (2018) found no significant relationship between RMET score and educational attainment, while other studies (Deng et al., 2022; Dodell-Feder et al., 2020; Schimit & Zachariae, 2009) found that years of schooling can explain some variance in the RMET score.

Familiarity with the four terms provided as potential response choices for each presented picture in the RMET is, presumably, a prerequisite for making the correct selection. It is reasonable to expect that persons with lower levels of education will have lower verbal intelligence and, thus, have greater difficulty achieving a high RMET score because they are less familiar with the presented terms. Moreover, the relative difficulty of the terms associated with each picture and the distinctiveness of the meanings of the four presented terms will vary across languages, so it is likely that the association of education level with the total RMET score (and with the pattern of incorrect RMET items) will vary for different language versions of the RMET. Assessment of item difficulty in each language (e.g. their frequency of use in daily speech) and comparison of RMET scores with measures of verbal intelligence will be needed to (1) decide on the minimum education level appropriate for administering the RMET; (2) develop a method of adjusting RMET scores based on education level or vocabulary skill, and (3) develop alternative versions of RMET suitable for persons with little formal education.

### Limitations

There are several potential limitations. (1) We only searched for studies published in English or Chinese, so the analyses did not include studies published in other languages or unpublished studies. (2) Some samples in the papers did not include data about key variables needed in the analysis (i.e. the standard deviation of mean RMET score, age of the sample, educational level of the sample) and some other studies were of low methodological quality. (3) Only 26 of the 198 studies directly compared RMET results of patients with schizophrenia and healthy controls, limiting our ability to conduct meta-analyses of results. (4) Most samples of patients with schizophrenia were chronic patients regularly using antipsychotic medications, so their deficits in theory of mind may not be representative of that in all individuals with schizophrenia. (5) The range in the mean age and mean years of education of the 40 samples of patients was relatively narrow, making it difficult to accurately assess the potential relation of age and education with RMET scores in the patients. (6) The distribution of the mean age of the 180 separate samples of healthy controls was imbalanced (the mean age of 88% of the samples was below 50), which potentially biased the assessment of the inflection point (at 32 years of age) in the meta-regression spline construction analysis. (7) Few studies reported other covariates of interest, including race, vocabulary level, and IQ participants; this made it difficult to explore the potential relationship of these variables with RMET performance in persons with schizophrenia.

## Conclusion

This is the first systematic review and meta-analysis of studies using the RMET to assess social cognitive functioning among individuals with schizophrenia. Meta-analyses of data from 198 identified studies confirm previous single-study findings that patients with schizophrenia experience severe impairments in theory of mind and, thus, support the construct validity of RMET. The consistency of these findings in multiple languages and several countries suggests that RMET may be a more cross-culturally valid measure of social cognition than other measures of social cognition like the MSCEIT that depend on respondents’ interpretation of social scenarios or vignettes. RMET scores decrease with age and increase with years of schooling in both patients and healthy controls, though these relationships were only statistically significant in the healthy control samples, possibly due to the much smaller number of patient samples available for analysis. The unexpectedly more significant differences between patients and controls when using non-English versions of the RMET than when using the original English version suggests that linguistic, racial, ethnic, and cultural differences also need to be considered when interpreting the results of the RMET. The assessed quality of most of the reports (based on a revised version of the STROBE reporting guidelines) was ‘fair’, and, interestingly, the quality of reports of studies using non-English versions of RMET was greater than that of studies using the original English version. In the multivariate meta-analysis of healthy control samples that included both age and years of schooling as covariates, years of schooling remained significantly associated with RMET scores, but age was no longer significantly associated with RMET scores. We also found a previously unreported non-monotonic relationship between age and RMET performance in healthy controls: the RMET score increased with age before age 31 and decreased with age after age 31. These findings highlight the need to clarify the relationships between age, education, verbal intelligence, and social cognition; they also suggest the need for a more nuanced assessment of the neurodevelopment of theory of mind – which may differ from the neurodevelopment of other cognitive abilities.

## Supplementary Material

supplement

**Supplementary material.** The supplementary material for this article can be found at https://doi.org/10.1017/S0033291723003501

## Figures and Tables

**Table 1.** Operational definition of eleven items used to assess the quality of the included studies

No.	Item content	*n* (%)^a^
1	Introduction provides rationale and specific objectives (hypothesis) for study.	197 (99.5%)
2	Method clearly describes the study design.	193 (97.5%)
3	Method section describes study setting(s), location, and date of recruitment.	22 (11.1%)
4	Diagnostic criteria, eligibility criteria, and recruitment process for individuals with schizophrenia and healthy controls are specified.	99 (50.0%)
5	Rationale for the sample size is provided.	10 (5.1%)
6	Describes all statistical methods used, and, if there is any missing data, how missing data is managed. (Assume no missing data if not mentioned.)	157 (79.3%)
7	Provides demographic characteristics of the sample that includes age and years of schooling.	126 (63.6%)
8	Reports numbers of individuals at each stage of study (e.g. numbers potentially eligible, examined for eligibility, confirmed eligible, included in the study, completing the assessment, and included in the analysis).	35 (17.7%)
9	Reports unadjusted mean of the number of correctly classified pictures (or % of correctly classified pictures) with standard deviation or confidence interval.	187 (94.4%)
10	Reports adjusted RMET score after controlling for age and years of schooling or reports the result of multivariate regression analyses using RMET score as the outcome variable that includes age and years of schooling as covariates.	3 (1.5%)
11	Discusses the limitations of the study.	163 (82.3%)

a number (percent) of the 198 studies included in the review that provide this information.

**Table 2.** Characteristics of 40 samples of patients with schizophrenia and 197 samples of healthy controls reported in the 198 included studies

Author (year)	Language of publication	Sample	Country	Language of RMET	Diagnostic criteria	Sample size	Mean (s.d.) RMET score	Quality score^a^
Baron-Cohen et al. (2001)	English	HC-A	US	English	NA	88	26.2 (3.6)	5
		HC-B	UK	English	NA	103	28.0 (3.5)	5
		HC-C	UK	English	NA	14	30.9 (3.0)	5
Craig (2004)	English	HC	UK	English	NA	16	27.6 (4.3)	3
Kelemen, Kéri, Must, Benedek, and Janka (2004)	English	HC	Hungary	Hungarian	DSM-IV	40	27.8 (5.0)	6
Havet-Thomassin, Allain, Etcharry-Bouyx, and Le Gall (2006)	English	HC	France	French	NA	17	27.6 (2.7)	5
Henry, Phillips, Crawford, Ietswaart, and Summers (2006)	English	HC	UK	English	NA	17	25.9 (4.1)	4
Meyer and Shean (2006)	English	HC	US	English	NA	142	28.9 (3.2)	5
Murphy (2006)	English	SCH	UK	English	ICD-10	13	19.0 (s.d. NA)	3
Bora, Sehitoglu, Aslier, Atabay, and Veznedaroglu (2007)	English	SCH	Turkey	Turkish	DSM-IV	58	18.2 (4.7)	8
Domes, Heinrichs, Michel, Berger, and Herpertz (2007)	English	HC	Germany	German	NA	30	25.0 (2.9)	3
Kemmis, Hall, Kingston, and Morgan (2007)	English	HC	UK	English	NA	21	26.4 (5.1)	5
Murphy (2007)	English	SCH	UK	English	NA	30	23.5 (6.1)	4
Slessor et al. (2007)	English	HC	UK	English	NA	40	24.0 (4.1)	3
Hirao et al. (2008)	English	SCH	Japan	Japanese	DSM-IV	20	19.2 (3.2)	7
		HC	Japan	Japanese	DSM-IV	20	24.3 (2.1)	7
Banati et al. (2009)	English	HC	Hungary	Hungarian	NA	35	25.2 (4.0)	4
Fertuck et al. (2009)	English	HC	US	English	DSM-IV	25	25.0 (3.6)	6
Harrison, Sullivan, Tchanturia, and Treasure (2009)	English	HC	UK	English	DSM-IV	20	30.6 (2.6)	5
Henry, Mazur, and Rendell (2009a)	English	HC	Australia	English	NA	30	28.7 (3.9)	5
Henry et al. (2009b)	English	HC	Australia	English	NA	20	32.1 (1.9)	4
Pardini and Nichelli (2009)	English	HC-A	US	Italian	NA	30	26.8 (2.8)	4
		HC-B	UK	Italian	NA	30	25.3 (3.2)	4
		HC-C	Italy	Italian	NA	30	23.5 (3.1)	4
		HC-D	Italy	Italian	NA	30	21.6 (2.2)	4
Rodrigues, Saslow, Gracia, John, and Keltner (2009)	English	HC	US	English	NA	179	27.4 (3.5)	3
Russel, Schmidt, Doherty, Young, and Tchanturia (2009)	English	HC	UK	English	NA	22	30.7 (1.9)	6
Schimit and Zachariae (2009)	English	HC	Denmark	Bosnian and Danish	NA	56	26.0 (3.2)	7
Smeets, Dziobek, and Wolf (2009)	English	HC	Netherlands and Germany	NA	NA	32	28.4 (1.1)	3
Couture et al. (2010)	English	SCH	US	English	DSM-IV	44	21.1 (5.7)	5
		HC	US	English	DSM-IV	41	25.0 (4.3)	5
de Achával et al. (2010)	English	SCH	Argentina	Spanish	DSM-IV	20	23.5 (5.4)	8
		HC	Argentina	Spanish	DSM-IV	20	27.3 (3.8)	8
Geraci, Surian, Ferraro, and Cantagallo (2010)	English	HC	Italy	Italian	NA	20	27.7 (4.1)	5
Gooding, Johnson, and Peterman (2010)	English	HC	US	English	NA	110	27.8 (3.0)	7
Kalbe et al. (2010)	English	HC	Germany	German	NA	28	25.6 (2.1)	5
Nietlisbach, Maercker, Rössler, and Haker (2010)	English	HC	Switzerland	German	NA	16	26.6 (2.8)	6
Lee, Guajardo, Short, and King (2010)	English	HC	US	English	NA	96	27.6 (3.5)	4
Oldershaw, Hambbook, Tchanturia, Treasure, and Schmidt (2010)	English	HC	UK	English	DSM-IV	47	28.4 (3.5)	9
Schimansky, Nicole, Rossler, and Haker (2010)	English	SCH	Switzerland	German	ICD-10	40	23.1 (3.2)	5
		HC	Switzerland	German	NA	39	25.5 (2.6)	5
Tso, Grove, and Taylor (2010)	English	HC	US	English	NA	33	26.4 (4.2)	7
Villa et al. (2010)	English	HC	US	English	NA	144	27.2 (3.3)	3
Zhang (2010)	Chinese	HC	China	Chinese	NA	126	20.4 (4.0)	4
Ahmed and Miller (2011)	English	HC	US	English	DSM-IV	123	27.3 (3.8)	5
Bai (2011)	Chinese	HC	China	Chinese	NA	118	22.1 (3.9)	4
Cavallo et al. (2011)	English	HC	UK	English	NA	21	27.0 (4.5)	5
Gooding and Pflum (2011)	English	HC	US	English	NA	68	27.2 (3.1)	6
Kim, Kwon, and Chang (2011)	English	HC	Korea	Korean	DSM-IV	27	25.6 (3.4)	7
Kornreich et al. (2011)	English	HC	Belgium	NA	DSM-IV-TR	25	23 (2)	6
Philippe et al. (2011)	English	HC	France	French	NA	18	26.3 (2.2)	6
Petroni et al. (2011)	English	HC	Argentina	Spanish	NA	16	26.7 (0.9)	4
Standford, Messinger, Malaspina, and Corcoran (2011)	English	HC	US	English	NA	14	27.9 (4.4)	7
Wolkenstein, Schönenberg, Schirm, and Hautzinger (2011)	English	HC	Germany	German	DSM-IV	20	23.6 (4.0)	6
Adenzato, Todisco, and Ardito (2012)	English	HC	Italy	Italian	NA	32	27.0 (3.3)	6
Cusi, MacQueen, and Mckinnon (2012)	English	HC	Canada	English	DSM-IV	25	26.2 (3.1)	7
Donohoe et al. (2012)	English	SCH	Ireland	English	DSM-IV	487	22.9 (5.7)	6
		HC	Ireland	English	NA	163	26.2 (4.2)	6
Geraci (2012)	English	SCH-A	Italy	Italian	DSM-IV	13	17.5 (5.0)	5
		SCH-B	Italy	Italian	DSM-IV	8	18.1 (2.1)	5
		SCH-C	Italy	Italian	DSM-IV	9	20.1 (4.4)	5
		HC	Italy	Italian	NA	20	27.7 (4.1)	5
Honekopp (2012)	English	HC	Germany	NA	NA	1896	24.4 (3.8)	4
Hysek, Domes, and Liechiti (2012)	English	HC	Switzerland	German	NA	48	24.4 (1.1)	8
Kenyon et al. (2012)	English	HC	UK	English	DSM-IV	57	28.3 (3.2)	7
Köther et al. (2012)	English	HC	Germany	German	MINI	30	25.5 (3.8)	7
Medina-Pradas, Navarro, Álvarez-Moya, Grau, and Obiols (2012)	English	HC	Spain	Spanish	DSM-IV-TR	39	27.2 (2.7)	7
Nejati, Zabihzadeh, Maleki, and Tehranchi (2012)	English	HC	Iran	Persian	DSM-IV	50	20.7 (2.8)	6
Pentaraki, Stefanis, Stahl, Theleritis, and Toulopoulou (2012)	English	SCH	Greece	Greek	DSM-IV-TR	21	19.1 (4.1)	3
Quintana, Guastella, Outhred, Hickie, and Kemp (2012)	English	HC	Australia	English	NA	65	27.4 (3.1)	7
Schilling et al. (2012)	English	HC	Germany	German	MINI	27	25.7 (3.9)	5
Szanto et al. (2012)	English	HC	US	English	DSM-IV	28	24.5 (5.1)	7
Barrera, Vázquez, Tannenhaus, Lolich, and Herbst (2013)	English	HC	Argentina	Spanish	DSM-IV/ICD-10	12	23.8 (3.0)	6
Buhlmann, Winter, and Kathmann (2013)	English	HC	Germany	German	NA	31	25.7 (3.4)	6
Caletti et al. (2013)	English	SCH	Italy	Italian	DSM-IV-TR	30	19.6 (4.6)	6
		HC	Italy	Italian	NA	18	25.7 (3.7)	6
De Los Reyes, Lerner, Thomas, Daruwala, and Goepel (2013)	English	HC	US	English	NA	50	25.2 (3.6)	5
De Sampaio, Soneira, Aulicino, and Allegri (2013)	English	HC	Argentina	Spanish	DSM-IV	24	25.9 (2.9)	7
Dehning et al. (2013a)	English	HC	Germany	English	NA	126	22 (4.3)	5
Dehning et al. (2013b)	English	HC	Germany and Ethiopia	English	NA	257	16.9 (S.D. NA)	4
Fernández-Abascal, Cabello, Fernández-Berrocal, and Baron-Cohen (2013)	English	HC	Spain	Spanish	NA	358	27.2 (3.6)	6
Giusti, Mazza, Pollice, Casacchia, and Roncone (2013)	English	SCH	Italy	Italian	NA	20	17.6 (5.9)	7
		HC	Italy	Italian	MINI	17	26.2 (2.9)	7
Pardini et al. (2013)	English	HC	Italy	Italian	NA	4150	24.6 (2.1)	7
Poletti and Bonuccelli (2013)	English	HC	Italy	Italian	NA	20	21.7 (3.0)	6
Poletti, Vergallo, Ulivi, Sonnoli, & Bonuccelli (2013)	English	HC	Italy	Italian	NA	35	22.1 (4.2)	5
Purcell, Phillips, and Gruber (2013)	English	HC	US	English	DSM-IV-TR	28	27.7 (S.D. NA)	5
Preller et al. (2013)	English	HC	Switzerland	German	DSM-IV	68	25.5 (3.8)	7
Thaler, Allen, Sutton, Vertinski, and Ringdahl (2013)	English	SCH	US	English	DSM-IV	30	18.7 (6.1)	8
		HC	US	English	DSM-IV	24	28.4 (2.8)	8
Thoma, Winter, Juckel, and Roser (2013)	English	HC	Germany	German	ICD-10	20	24.5 (4.0)	8
Whitton and Henry (2013)	English	SCH	Australia	English	DSM-IV	34	24.0 (5.7)	3
		HC	Australia	English	NA	44	26.7 (4.7)	3
Balogh, Égerházi, Berecz, and Csukly (2014)	English	SCH	Hungary	Hungarian	DSM-IV	43	17.9 (5.2)	6
		HC	Hungary	Hungarian	NA	41	24.1 (3.8)					
Bedwell et al. (2014)	English	HC	United States	English	DSM-IV-TR	686	24.0 (4.3)	7
Csukly, Polgár, Tombor, Benkovits, and Réthelyi (2014)	English	HC	Hungary	Hungarian	DSM-IV	29	25.3 (3.7)	6
		SCH-A	Hungary	Hungarian	DSM-IV	30	19.1 (4.9)	6
		SCH-B	Hungary	Hungarian	DSM-IV	28	21.3 (5.2)	6
Dal Monte et al. (2014)	English	HC	US	English	NA	29	25.2 (0.6)	7
Demartini et al. (2014)	English	HC	UK	English	DSM-IV	34	24.2 (3.9)	6
Goddard, Carral-Fernández, Denneny, Campbell, and Treasure (2014)	English	HC	UK	English	NA	42	26.8 (4.0)	6
Gong, Liu, Li, and Zhou (2014)	English	HC	China	Chinese	NA	322	21.2 (4.0)	5
Lam, Raine, and Lee (2014)	English	SCH	Hong Kong, China	Chinese	DSM-IV	58	19.1 (4.1)	8
		HC	Hong Kong, China	Chinese	NA	61	22.7 (3.1)	8
Montag et al. (2014)	English	SCH-A	Germany	German	DSM-IV	16	20.6 (5.5)	9
		SCH-B	Germany	German	DSM-IV	19	20.7 (5.9)	9
Nandrino et al. (2014)	English	HC	France	French	NA	30	24.0 (3.3)	6
Prevost et al. (2014)	English	HC-A	Canada	French	NA	127	24.8 (3.8)	6
		HC-B	Canada	French	NA	139	25.6 (5.4)	6
Reynolds, Van Rheenen, and Rossell (2014)	English	HC	Australia	English	DSM-IV-TR	20	28.8 (2.7)	5
Woolley et al. (2014)	English	HC	US	English	DSM-IV	31	26.2 (3.6)	7
Youssef, Nunes, Sa, and Williams . (2014)	English	HC	West Indies	NA	NA	655	25.7 (3.9)	4
Ainley, Maister, and Tsakiris (2015)	English	HC	UK	English	NA	97	26.6 (6.1)	4
Baron-Cohen et al. (2015)	English	HC-A	US	English	DSM-IV or ICD-10	152	25.5 (4.6)	7
		HC-B	UK	English	DSM-IV or ICD-10	168	27.4 (3.4)	7
Boucher et al. (2015)	English	HC	Canada	French	NA	20	26.2 (3.6)	7
Cabinio et al. (2015)	English	HC	Italy	Italian	NA	36	26 (3.9)	4
Carré et al. (2015)	English	HC	Canada	English	NA	30	26.0 (3.5)	6
Eddy and Rickards (2015)	English	HC	UK	English	NA	26	26.7 (3.1)	6
Enrici et al. (2015)	English	HC	Italy	Italian	NA	25	23.4 (4.7)	5
Fisher et al. (2015)	English	HC	Italy	Italian	DSM-5	43	25.6 (4.5)	7
Jelson-Swain, Persad, Burkard, and Welsh (2015)	English	HC	US	NA	NA	17	25.4 (2.3)	8
Launay et al. (2015)	English	HC-A	US	English	NA	279	26.9 (4.8)	6
		HC-B	UK	English	NA	26	25.9 (6.2)	6
Lawson (2015)	English	HC	UK	English	NA	152	26 (3.8)	3
Ma, Guo, Zhang, and He (2015)	Chinese	HC	China	Chinese	NA	95	23.7 (4.7)	6
Melchers, Montag, Markett, and Reuter (2015)	English	HC	Germany	German	NA	108	24.6 (2.5)	6
Moieni, Irwin, Jevtic, Breen, and Eisenberger (2015)	English	HC	US	English	DSM-IV	109	28.2 (0.5)	9
Radke and de Bruijn (2015)	English	HC	Netherlands	Dutch	NA	24	24.4 (4.5)	7
Robinson, Gary, Burt, Ferrier, and Gallagher (2015)	English	HC	UK	English	NA	28	26.8 (3.5)	3
Scherzer et al. (2015)	English	SCH	Canada	French	DSM-IV	21	20.2 (2.9)	7
		HC	Canada	French	NA	29	21.7 (4.0)	7
Schuwerk, Vuori, and Sodian (2015)	English	HC	Germany	German	NA	17	25.5 (3.2)	5
Tella et al. (2015)	English	HC	Italy	Italian	NA	41	26.8 (3.6)	6
Weisman et al. (2015)	English	HC	Singapore	English	NA	1463	25.9 (4.3)	5
Zilioli, Ponzi, Henry, and Maestripieri (2015)	English	HC	US	English	NA	469	26.8 (3.5)	6
Ayesa-Arriola et al. (2016)	English	HC	Spain	Spanish	NA	159	23.8 (4.3)	8
Besnard et al. (2016)	English	HC	France	French	NA	30	21.3 (3.9)	6
Brambilla et al. (2016)	English	SCH	Italy	Italian	DSM-IV	32	20.8 (4.8)	9
Bora, Veznedzroglu, and Vahip (2016)	English	SCH	Turkey	Turkish	DSM-IV	54	14.2 (3.9)	6
		HC	Turkey	Turkish	NA	27	19.4 (2.6)	6
Burke et al. (2016)	English	HC	Ireland	English	NA	50	25.2 (4.9)	6
El Haj et al. (2016)	English	HC-A	France	French	NA	40	26.8 (4.5)	6
		HC-B	France	French	NA	36	22.8 (4.5)	6
Filippo et al. (2016)	English	HC	Italy	Italian	NA	20	28 (5.0)	5
Haag, Haffner, Quinlivan, Brüne, and Stamm (2016)	English	HC	Germany	German	NA	29	23.6 (4.0)	8
Heitz et al. (2016)	English	HC	France	French	NA	16	23.9 (2.8)	7
Hoche, Guell, Sherman, Vangel, and Schmahmann (2016)	English	HC	US	English	NA	57	27.9 (3.3)	5
Javkowiak-Siuda et al. (2016)	English	HC	Poland	Polish	NA	325	25.0 (4.5)	5
Jermakow and Brzezicka (2016)	English	HC	Poland	Polish	NA	60	24.8 (4.3)	6
Melchers, Montag, Reuter, Spinath, and Hahn (2016)	English	HC	Germany	German	NA	716	23.0 (3.3)	5
Oldershaw et al. (2016)	English	HC	Poland	Polish	NA	47	25.7 (4.1)	7
Petersen, Brakoulia, and Langdon (2016)	English	HC	Australia	English	DSM-IV	20	29.1 (3.2)	8
Pino et al. (2016)	English	HC	Italy	Italian	DSM-IV	23	24.4 (6.3)	6
Rominger et al. (2016)	English	HC	Austria	German	NA	20	23.5 (3.9)	4
Sabater et al. (2016)	English	HC	Spain	Spanish	NA	25	25.6 (2.4)	5
Schneider et al. (2016)	English	HC	US	English	NA	65	28.0 (4.1)	6
Wang, Song, Zhen, and Liu (2016)	English	HC	China	Chinese	NA	268	23.6 (3.5)	6
Bodnar and Rybakowski (2017)	English	HC	Poland	Polish	NA	25	28.1 (3.4)	6
Charernboon and Patumanond (2017)	English	SCH	Thailand	Thai	DSM-5	36	18.9 (4.4)	10
		HC	Thailand	Thai	NA	36	23.5 (4.4)	10
Chen et al. (2017)	English	SCH	Taiwan	Traditional Chinese	DSM-5	53	19.5 (3.4)	7
DeAngelo and McCannon (2017)	English	HC	US	English	NA	141	21.7 (3.7)	2
Dulau et al. (2017)	English	HC	France	French	NA	65	24.0 (3.2)	7
Enrici et al. (2017)	English	HC	Italy	Italian	NA	20	24.2 (4.7)	5
Erdeniz, Serin, Íbadi, and Tas (2017)	English	SCH	Turkey	Turkish	DSM-IV-TR	23	16.0 (5.6)	6
		HC	Turkey	Turkish	NA	23	24.5 (3.9)	6
Gavilá and Haro (2017)	English	HC	Spain	English	NA	95	25.1 (3.3)	5
Helle, Løberg, Gjestad, Schnakenberg Martin, and Lysaker (2017)	English	SCH	US	English	DSM-IV	87	21.1 (5.4)	7
Hotier et al. (2017)	English	HC	France	French	NA	36	24.4 (s.d. NA)	5
Leppanen et al. (2017)	English	HC	UK	English	DSM-5	29	25.8 (3.5)	8
Martinez-Sanchez, Fernández-Abscal, and Sánchez-Pérez (2017)	English	HC	Spain	Spanish	NA	1398	26.8 (3.4)	6
Newbury-Helps, Feigenbaum, and Fonagy (2017)	English	HC	UK	English	NA	42	25.7 (0.8)	5
Preti, Vellante, and Petretto (2017)	English	HC	Italy	Italian	NA	200	24.8 (4.2)	6
Raimo et al. (2017)	English	HC	Italy	Italian	DSM-5	40	29.0 (0.6)	7
Sanvicente-vieira, Kluwe-schiavon, Corcoran, and Grassi-Oliveira (2017)	English	HC	Brazil	Portuguese	NA	30	25.1 (2.7)	7
Sun et al. (2017)	Chinese	HC	China	Chinese	NA	49	23.2 (4.1)	5
Tylec, Jeleniewicz, Mortimer, Bednarska-Makaruk, and Kucharska (2017)	English	HC	Poland	Polish	NA	50	25.7 (5.2)	7
Zabihzadeh et al. (2017)	English	HC	Iran	Persian	DSM-IV	25	24.9 (4.8)	7
Anupama, Poornima, Jagadisha, and Urvakhsh (2018)	English	HC	India	Kannada	NA	20	28.9 (2.0)	5
Atoui et al. (2018)	English	SCH	Lebanon	Lebanese	DSM-5	22	22.4 (5.8)	7
		HC	Lebanon	Lebanese	NA	21	26.6 (2.6)	7
Aydin, Lysaker, Balıçı, Ünal-Aydin, and Esen-Danaci (2018)	English	SCH	Turkey	Turkish	DSM-IV-TR	34	16.5 (4.5)	6
		HC	Turkey	Turkish	NA	31	20.7 (4.8)	6
Berenson et al. (2018)	English	HC	US	English	DSM-IV	60	27.7 (0.5)	6
Coyle, Elpers, Gonzalez, Freeman, and Baggio (2018)	English	HC	US	English	NA	249	27.6 (3.5)	5
Eddy, Rickards, and Hansen (2018)	English	HC	UK	English	NA	28	27.4 (0.6)	6
Espinós, Fernández-Abascal, and Ovejero (2018)	English	HC	Spain	Spanish	DSM-IV-R	112	27.4 (3.2)	6
Fossati, Borroni, Dziobek, Fonagy, and Somma (2018)	English	HC	Italy	Italian	NA	193	24.9 (3.9)	5
Grainger, Henry, Naughtin, Comino, and Dux (2018)	English	HC	Australia	English	NA	50	25.9 (1.4)	6
Khorashad et al. (2018)	English	HC	Iran	Persian	DSM-V	104	22.3 (2.7)	7
Lee et al. (2018)	English	SCH	US and Korea	Korean	DSM-IV	60	20.9 (5.4)	9
		HC	US and Korea	Korean	DSM-IV	60	26.7 (4.0)	9
Navarra-Ventura et al. (2018)	English	HC	Spain	Spanish	NA	40	29.0 (2.4)	6
Lopez-Navarro (2018)	English	SCH	Spain	Spanish	DSM-IV-TR	54	17.4 (4.5)	5
Pinkham, Harvey, and Penn (2018)	English	HC	US	English	NA	154	24.8 (4.3)	7
Redondo and Herrero-Fernández (2018)	English	HC	Spain	Spanish	NA	433	15.4 (2.2)	5
Uhlmann, Ipser, Wilson, and Stein (2018)	English	HC	South Africa	English	DSM-IV	21	24.1 (5.0)	7
Wang (2018)	Chinese	SCH-A	China	Chinese	DSM-5	24	18.9 (4.8)	10
		SCH-B	China	Chinese	DSM-5	22	17.2 (6.4)	10
Yang, Khalifa, Lankappa, and Völlm (2018)	English	HC	UK	NA	NA	23	24.6 (4.4)	7
Adenzato et al. (2019)	English	HC	UK	English	NA	20	22.9 (3)	6
Altunbas, Unsalver, and Yasar (2019)	English	HC	Turkey	Turkish	NA	30	31.0 (2.9)	5
Bayliss et al. (2019)	English	HC	Mexico	Spanish	NA	12	24 (2.7)	5
Black (2019)	English	HC	US	English	NA	591	26.3 (5.3)	5
Budak, Küçükgöncü, and Bestepe (2019)	English	HC	Turkey	Turkish	NA	60	25.6 (3.9)	7
Dalkner et al. (2019)	English	HC	Austria	German	NA	79	23.0 (4.2)	5
Duque-Alarcón, Alcalá-Lozano, González-Olvera, Garza-Villarreal, and Pellicer (2019)	English	HC	Mexico	Spanish	NA	15	25.7 (4.3)	6
Giordana et al. (2019)	English	HC	Mexico	Spanish	NA	116	25.6 (3.8)	6
Hartman, Heinrichs, and Mashhadi (2019)	English	SCH	Canada	English	DSM-IV-TR	44	21.2 (5.4)	7
		HC	Canada	English	DSM-IV-TR	62	25.2 (5.4)	7
Lopez-del-Hoyo et al. (2019)	English	SCH	Spain	Spanish	DSM-IV or ICD-10	30	13.1 (6.7)	7
		HC	Spain	Spanish	NA	30	26.6 (4.7)	7
Romosan et al. (2019)	English	HC	Romania	Romanian	NA	51	24.9 (4.6)	6
Sacchetti et al. (2019)	English	HC	UK	English	NA	51	28.3 (3.2)	8
Simon et al. (2019)	English	HC	Hungry	Hungarian	NA	32	26.7 (3.1)	7
Tapajóz, Soneira, Catoira, Aulicino, and Allegri (2019)	English	HC	Argentina	Spanish	NA	17	26.5 (3.0)	6
Zegarra-Valdivia and Vilca (2019)	English	HC	Perú	Spanish	DSM-5	20	24.0 (3.3)	4
Charernboon and Patumanond (2020)	English	SCH	Thailand	Thai	DSM-5	50	19.1 (4.0)	8
		HC	Thailand	Thai	DSM-5	50	23.4 (4.1)	8
Cotelli et al. (2020)	English	HC-A	Italy	English	NA	32	24.3 (0.5)	5
		HC-B	Italy	English	NA	30	22.1 (0.4)	5
Eddy and Hansen (2020)	English	HC	UK	English	NA	176	25.5 (1.5)	7
Ignatova et al. (2020)	English	HC	India	NA	NA	36	24.1 (3.9)	5
Kéri, Kállai, and Csigó (2020)	English	SCH	Hungary	Hungarian	DSM-5	32	19.1 (4.3)	6
		HC	Hungary	Hungarian	NA	32	23.6 (4.9)	6
Kilic et al. (2020)	English	HC	Turkey	Turkish	DSM-IV-TR	35	23.2 (2.6)	7
McPhilemy et al. (2020)	English	HC	Ireland	English	DSM-V-TR	49	26.5 (3.8)	6
Pahnke, Mau-Moeller, Hamm, and Lischke (2020)	English	HC	Germany	German	NA	119	25.6 (0.4)	5
Ünal-Aydin, Balıçı, Sönmez, and Aydin (2020)	English	HC	Sarajevo, Bosnia and Herzegovina	NA	NA	337	23.3 (3.3)	7
García-Fernández, Cabot-Ivorra, Romero-Ferreiro,Pérez-Martín, and Rodriguez-Jimenez (2020)	English	SCH-A	Spain	Spanish	DSM-5	43	23.7 (4.4)	8
		HC-A	Spain	Spainish	MINI	50	26.7 (3.3)	8
		SCH-B	Spain	Spanish	DSM-5	44	20.4 (4.8)	8
		HC-B	Spain	Spainish	MINI	52	24.8 (4.8)	8
Wilu Wilu, Allain, Moustafa, and El Haj (2019)	English	HC	France	French	NA	28	27.5 (3.7)	5

HC, healthy controls; SCH, patients with schizophrenia; NA, not available.

a Quality score assessed by study authors based on 11 items listed in Table 1 (total score ranges from 0 to 11).

**Table 3.** Meta-regression of RMET score on age and years and schooling

	Covariates	Individuals with schizophrenia			Healthy controls		
		Number of samples	Coef	*p* value	Number of samples	Coef	*p* value*
Model 1	Age	40	−0.045	0.516	180	−0.031	**0.020**
Model 1 using bootstrap			−0.045	0.527		−0.031	**0.018**
Model 2	Years of schooling	35	0.399	0.149	98	0.477	**<0.001**
Model 2 using bootstrap			0.399	0.076		0.477	**<0.001**
Model 3	Age	35	−0.032	0.635	99	−0.026	0.126
	Years of schooling		0.418	0.140		0.423	**<0.001**
	Constant		15.88	**<0.001**		20.79	**<0.001**
Model 3 using bootstrap	Age		−0.032	0.648		−0.026	0.106
	Years of schooling		0.418	0.081		0.423	**<0.001**
	Constant		15.88	**<0.001**		20.80	**<0.001**

* *p*-values printed in bold indicated that the result is statistically significant.

**Table 4.** Relationship of age and RMET score among healthy controls using univariate and multivariate meta-regression with spine construction

Covariates	Spine cut-off	Coefficient	*p* value*
Age only (number of samples = 180)	$\frac{dRMET}{dage}|\text{age}⩽31$	0.123	**0.008**
	$\frac{dRMET}{dage}|\text{ae}>3$	−0.074	**<0.001**
	constant	22.59	**<0.001**
Age and years of schooling (number of samples = 99)	$\frac{dRMET}{dage}|\text{age}⩽31$	0.179	**0.048**
	$\frac{dRMET}{dage}|\text{ae}>3$	−0.048	**0.011**
	Years of schooling	0.427	**<0.001**
	constant	14.79	**<0.001**

* *p*-values printed in bold indicated that the result is statistically significant.
