# Supplementary material for: Assessing social cognition in patients with schizophrenia and healthy controls using the reading the mind in the eyes test (RMET): a systematic review and meta-regression
Source: Psychol Med. Author manuscript; Available in PMC 2026 Jun 22. (PMC13285861; doi:10.1017/S0033291723003501)
Supplement: supplement [file NIHMS2181666-supplement-supplement.pdf]

Assessing social cognition in patients with schizophrenia and healthy controls using the Reading the Mind in the Eyes Test (RMET): a systematic review and meta-regression

Deng et al.

Supplementary materials

APPENDIX ONE: Search strategies in different databases

Pubmed: "RMET"[All Fields] OR (("reading"[MeSH Terms] OR "reading"[Title/Abstract] OR "readings"[Title/Abstract]) AND ("sci am mind"[Journal] OR "mind"[Journal] OR "mind"[All Fields]) AND ("eye"[MeSH Terms] OR "eye"[Title/Abstract] OR "eyes"[Title/Abstract])) OR (((("schizophrenia"[MeSH Terms] OR "schizophrenia"[Title/Abstract] OR "schizophrenias"[Title/Abstract] OR "schizophrenia s"[Title/Abstract]) AND (("eye"[MeSH Terms] OR "eye"[Title/Abstract]) AND ("research design"[MeSH Terms] OR ("research"[Title/Abstract] AND "design"[Title/Abstract]) OR "research design"[Title/Abstract] OR "test"[Title/Abstract]))) NOT "RMET"[Title/Abstract]) NOT (("reading"[MeSH Terms] OR "reading"[Title/Abstract] OR "readings"[Title/Abstract]) AND ("sci am mind"[Journal] OR "mind"[Journal] OR "mind"[Title/Abstract]) AND ("eye"[MeSH Terms] OR "eye"[Title/Abstract] OR "eyes"[Title/Abstract])))
Search date: 6th Aug. 2020

Web of science: (AB=RMET) OR (AB =reading the mind in the eyes) OR (AB=reading the mind in the eye)OR ((AB =schizophrenia) AND (AB =eye test) NOT ((AB =RMET) OR (AB =reading the mind in the eyes) OR (AB=reading the mind in the eye)))
Search date: 6th Aug. 2020

Psycinfo/EBSCO: AB RMET OR AB reading the mind in the eyes OR AB reading the mind in the eye OR (AB schizophrenia) AND (AB eye test) NOT ((AB RMET) OR (AB reading the mind in the eyes) OR (AB reading the mind in the eye)))
Search date: 6th Aug. 2020

CNKI: TKA=RMET OR TKA=读眼识心 OR TKA=读眼阅读 OR TKA=眼区读心 OR TKA=眼神阅读 OR TKA=Reading the Mind in the Eyes OR TKA=reading the Mind in the Eye OR (TKA=schizophrenia AND (TKA=眼区 OR TKA=眼神) NOT TKA=RMET NOT TKA=reading the mind in the eyes NOT TKA=reading the mind in the eye)
Search date: 11th Aug. 2020

Wanfang: 摘要:(RMET) or 摘要:('读眼识心') or 摘要:('读眼阅读') or 摘要:('眼区读心') or 摘要:('眼神阅读') or 摘要:('Reading the Mind in the Eyes') or 摘要:('reading the Mind in the Eye') or (摘要:(schizophrenia) and (摘要:('眼区') or 摘要:('眼神')) not 摘要:(RMET) not 摘要:('reading the mind in the eyes') not 摘要:('reading the mind in the eye'))
Search date: 11th Aug. 2020
